# Effect of a Simulation-Based Handover Education Program for Nursing Students: A Quasi-Experimental Design

**DOI:** 10.3390/ijerph18115821

**Published:** 2021-05-28

**Authors:** Da-Hye Lee, Eun-Ju Lim

**Affiliations:** 1 Seoul National University Bundang Hospital, 172, Dolma-ro, Bundang-gu, Seongnam-si 13605, Korea; dahae8273@naver.com; 2 Red Cross College of Nursing, Chung-Ang University, 84, Heukseok-ro, Dongjak-gu, Seoul 06974, Korea

**Keywords:** clinical competence, handover knowledge, nursing education, nursing student, patient handover, patient simulation, self-efficacy

## Abstract

Nursing handover facilitates the continuity of nursing and ensures patient safety and quality of care. This study aimed to evaluate the effectiveness of a handover education program by assessing handover knowledge, self-efficacy, and handover performance competency. A group pretest–post-test quasi-experimental design was used. Thirty 4th-year Korean nursing students participated in a handover education program comprising a lecture and simulation training using a high-fidelity simulator. The average level of handover knowledge was 4.63 ± 1.61 before the program and 5.83 ± 0.95 after (t = −3.71, *p* = 0.001). Meanwhile, the average self-efficacy score was 3.35 ± 0.57 before the program and 3.90 ± 0.60 after (t = −5.65, *p* < 0.001). Further, the average handover performance competency was 1.75 ± 0.25 before the program and 2.37 ± 0.21 after (t = −12.08, *p* < 0.001). The simulation-based handover education intervention was effective in improving knowledge, self-efficacy, and performance competency of nursing students. This intervention can provide an effective method of improving nursing students’ handover skills prior to entering clinical practice.

## 1. Introduction

Handover is a communication process among nurses that involves conveying patient information and is important in ensuring quality and continuity of nursing care [1,2]. Accurate and comprehensive handover helps nurses sufficiently understand patient needs and increases the stability and efficacy of nursing; it also has a positive impact on nurses’ job satisfaction and enhancing their expertise [3]. Accurate handovers help reduce errors and enable nurses who lack clinical experience to improve by receiving suggestions from advanced nurses [4]. However, some inexperienced nurses often make mistakes in the handover process for various reasons, such as their lack of nursing experience, psychological crisis, and relationship conflicts, all of which can hinder the effectiveness of nursing work [4]. Such errors also delay the diagnosis, treatment, and evaluation of patients, thereby increasing patient morbidity and mortality, and possibly longer-term hospitalization, reducing patient satisfaction [5,6]. Thus, studies reporting the current state of handover by clinical nurses in Korea constantly emphasize the need for educating nurses on accurate handover methods [4,7,8].

During handover, team members inspect patients’ treatment plans and reorganize the nursing diagnoses [9]. Handover requires comprehensive judgment and communication about the clinical situation related to a patient’s disease, thereby directly affecting patient safety. However, there are limitations that affect handover training among nursing students. In practice, their handover performance shows only minor improvement in clinical training, which is focused on observation [10]. Thus, nursing students must have opportunities to understand patients’ conditions thoroughly and deliver the details of nursing practice through handover training [11]. To this end, the undergraduate nursing course must have an educational program on handover [11,12]. In [13], 62.6% of nurses were found to have no experience in handover. Moreover, most instances of handover education for new nurses consisted of merely observing the handovers of fellow nurses or learning from their senior nurses through simple oral instructions [7]. This has resulted in reported difficulties on the handover performance of new nurses for assigned patients [12]. Therefore, it is necessary to improve the current nursing education system and develop education programs to bridge this gap in nurses’ training [13,14].

This can be accomplished with simulation-based programs, which allow students to experience the problem-solving process while gaining theoretical knowledge. It also helps them practice in a realistic setting, thereby improving their communication skills and self-efficacy [15]. Further, it improves their knowledge, confidence, psychomotor skills, and critical thinking skills [16] and is more effective than the traditional training method [17]. Studies have indicated that handover programs improved students’ communication skills and handover performance competency when combined with lectures, group discussions, role-playing, and simulation-based education [18,19]. Although the best method for handover programs is not clearly stated, simulation-based training and role-playing are widely used to improve handover competency [20].

Handover is not merely the delivery of information about the assessment of a patient’s condition, but a dynamic and complex task that includes clinical judgment and evaluation of the patient’s condition [21]. For nursing students to accomplish their learning objectives and acquire necessary competencies, it is important to provide them with opportunities to think critically about what to assess, integrate, and judge during handovers [22]. Gaining this experience through simulation-based programs that can reproduce various scenarios is an effective way to achieve this goal [23]. Accordingly, this study aimed to develop, apply, and verify the effectiveness of a simulation-based handover education program for senior-year nursing students. The findings could help nursing students adapt to nursing practice after graduation. The handover experience acquired through scenario-based clinical settings provides basic data for theoretical and practical training to improve nursing students’ handover skills in the future.

## 2. Materials and Methods

### 2.1. Research Design

This study used a one-group pretest–post-test design to develop and implement a simulation-based handover education program and verify its effects.

### 2.2. Participants

This study was conducted on senior students attending a nursing college in Korea, who showed interest in simulation-based handover education after listening to the objectives of the study and voluntarily agreeing to participate. Sample size was calculated using G*Power 3.1.9.2 [24], and the effect size was calculated as medium 0.50, with significance level 0.05 and statistical power (1-β err prob) 0.80. The power is the probability that when an alternative hypothesis is true, it will be determined as fact. As the power improves, the probability of a type 2 error decreases. The minimum number of participants required was 27; ultimately, 30 participants were extracted by convenience sampling considering the dropout rate. The data of all 30 subjects were analyzed.

### 2.3. Data Collection

To develop a handover education program and verify its effects, this study recruited research participants after obtaining permission from one of the colleges of nursing in Korea. Data were collected from 15 February to 2 March 2018.

### 2.4. Instruments

#### 2.4.1. Handover Knowledge

Handover knowledge was measured using a tool designed based on the Korean Nurses Association’s refresher training “Integrative Application of the Situation Background Assessment Recommendation (SBAR) Communication Method” [25] and a previous study on developing handover practice standards and standardized items for nurses in Korean hospitals [8]. The questionnaire assessing handover knowledge comprised multiple-choice questions with four options, wherein 0 was assigned for an incorrect answer and 1 for a correct answer. The scores ranged from 0 to 7 points, with higher scores indicating a higher level of handover knowledge. Item discrimination was analyzed as the top 27% and bottom 27%, based on the total score of the test [26]; it was found that all seven items had a discrimination power with discrimination index >0.2.

#### 2.4.2. Self-Efficacy

Self-efficacy was measured using a self-administered questionnaire developed by Sherer et al. [27], modified by Jung [28], and then modified and improved by the researcher for this study. This tool consists of 17 items rated on a 5-point scale ranging from “Strongly agree” (5 points) to “Strongly disagree” (1 point). The total scores range from 17 to 85 points, with higher scores indicating higher self-efficacy. The reliability of the tool was verified based on Cronbach’s ⍺ of 0.94 in the study by Jung [28] and 0.958 in this study.

#### 2.4.3. Handover Performance Competency

Handover performance competency was measured using the Patient, Assessment, Situation, Safety concerns, Background, Action, Recommendation method (PASS-BAR) from previous research [8] that studied standardized items of handover and was reorganized by the researchers according to the scenarios in this study. Content validity was verified by a nursing professor, two managers (chief nurse or higher), and two nurses with 10 years of experience. The tool was then modified and improved, ultimately using 15 items. The handover performance competency scorecard was categorized into the PASS-BAR format and restructured according to the scenarios. There were 15 items in all: 1 item on the patient’s general situation, 5 items on patient assessment, 1 item on safety concerns, 3 items on background, 3 items on action, and 2 items on recommendation. Each item is rated on a 3-point scale based on whether the item is not performed, partially performed, or adequately performed: “Not performed” (1 point), “Partially performed” (2 points), and “Adequately performed” (3 points). The total scores ranged from 15 to 45, with higher scores indicating higher handover performance competency. In this study, Cronbach’s ⍺ was 0.727.

### 2.5. Research Procedure

The simulation-based handover education program comprised five steps: analysis, design, development, implementation, and evaluation based on the Analysis, Design, Development, Implementation, and Evaluation (ADDIE) model [29], a typical model for instructional system design.

### 2.6. Analysis

#### 2.6.1. Analysis of Educational Needs

A survey on educational needs was conducted on 40 senior nursing students at S University. Nearly 72.2% of the students considered handover “very important” in nursing practice, while 19.4% said it is “important”. Further, ~55.6% responded that the program is “highly necessary”, while 33.3% responded that it is “necessary.” All the students (100%) claimed that they had no educational experience in handover. Simulation training was the most highly rated method, with 56.6% saying it was the best “method of education” to improve handover performance. Handover based on changes in the patient’s condition, as the “area most requiring” a handover program, was rated highest at 75%, while the “appropriate time for the program” was in the senior year, according to 52.8% of the students.

#### 2.6.2. Analysis of the Educational Environment

For an enhanced sense of realism in the general ward, research was conducted in the simulation-center training room of S University in Gyeonggi. Based on the scenario, the venue was equipped with a high-fidelity simulator (Sim-Man, Laerdal Medical, Wappingers Falls, NY, USA) as well as audio and video devices to implement a real-life scenario. The handover took place in the nurses’ station.

### 2.7. Design

#### 2.7.1. Topic and Goals of Education

Nursing handover was conducted for a patient with respiratory problems. This was done based on the results of analyzing participants’ educational needs, and the specific goals of the learning were divided into knowledge, skills, and attitude.

#### 2.7.2. Design of the Evaluation Tool

To evaluate the effects of the simulation-based handover education program, handover knowledge and self-efficacy before and after the education program were evaluated by the subjects themselves through a self-administered questionnaire. Handover performance competency was evaluated by the researcher before and after the education program.

### 2.8. Development

#### 2.8.1. Scenario Development

Two scenarios involving patients with respiratory problems were developed using the high-fidelity simulator. Scenarios I and II were developed with reference to an actual clinical case to implement the handover situation, according to the changes in the patients’ conditions, and were used in the evaluation before and after education. The same algorithm was used to maintain a consistent level of difficulty in the scenarios. Moreover, an overview of the situation, guidelines, and debriefing plans for the students were developed, as well as an outline of the cases and a checklist for the professors.

#### 2.8.2. Verification of Content Validity

The contents of the scenarios were inspected by two nursing professors and three clinical nurses with at least 10 years of experience, to determine whether they were realistic and could be seen in a real clinical situation. There were additional requests in the verification process of Scenario I, such as administration of antihypertensive drugs (+) and findings of Computed Tomography. There was also additional advice on allergies (+) in the verification process for Scenario II, which was revised and inspected a second time before ultimately being used in the program.

#### 2.8.3. Pilot Test

To identify and resolve issues before implementing the simulation-based handover education program, and to check the scenarios’ suitability and running time, supplies for preparation, and unexpected problems, a pilot test was conducted with two nursing students who did not participate in the research.

### 2.9. Implementation

The handover education program was implemented from 19 February to 2 March 2018, over 8 sessions, with 120 min of education per student. Students were divided into groups of 3–4 for 120-min sessions that consisted of 50 min of theoretical education and 70 min of simulation-based education.

#### 2.9.1. Theoretical Education

Theoretical education on handover included its definition and importance, practical standards and guidelines, current status, contents, and methods of handover. The importance was explained by presenting cases of inappropriate handovers, and methods were explained by the order and items involved in handover. A PowerPoint presentation was used, and an open discussion followed.

#### 2.9.2. Simulation-Based Education

The scenarios were run one by one in the simulation-based component of the program, consisting of pre-briefing and running the simulation. During the pre-briefing, the participants learned the order of the simulation, setting, supplies for preparation, and the starting conditions of the scenarios. They were then given enough time to familiarize themselves with the environment and check the location and use of supplies. The participants listened to the researcher’s explanation about the contents and beginning of the scenarios, then experienced the high-fidelity simulator. A nurse with 10 years of experience, who was trained with simulation-based education, played the doctor’s role and served as the operator. The nursing students assessed the patients according to the changes in their condition and communicated with the doctor by delivering an oral report over the phone. They then performed nursing care according to these changes, and determined and combined the contents of handover in the evaluation step of the nursing process. The receiver of the handover was an experienced nurse in the next shift. After completing the simulation, the students participated in a 40 min debriefing in 3–4 people per group. Debriefing was conducted by designing questions for the description, analysis, and application phases, as presented by Fanning and Gaba [30]. In the description phase, the participants were asked to reflect on the scenario structure and situation, summarize the overall training situation, and identify topics of discussion. In the analysis phase, they talked about their emotions while experiencing handover, as well as their strengths and weaknesses, and discussed how to improve their performance. In the application phase, they looked back on what they learned from the simulation-based education and summed up the results to apply them to actual clinical practice.

### 2.10. Evaluation

Evaluation was performed the same way as the pre-evaluation to verify the effects of the simulation-based handover education program.

### 2.11. Ethical Considerations

Ethical approval was obtained from the Institutional Review Board (IRB File No: 1041078-201712-HR-243-01) prior to conducting this study. Nursing students expressing an interest in participating were informed about the study by the lead researcher, and written consent was obtained for participation.

### 2.12. Data Analysis

All statistical analyses were performed using SPSS version 22.0 (SPSS Inc., Chicago, IL, USA). Descriptive statistical analysis was used to classify participants’ general characteristics. Handover knowledge, self-efficacy, and handover performance competency of participants, before and after participating in the simulation-based handover education program, were analyzed using a paired *t*-test.

## 3. Results

### 3.1. Participants’ General Characteristics

The average age of the participants was 22.17 years, and all 30 of them were female. The students required 90 h of clinical practice per subject; 25 (83.3%) participants had clinical practice experience in 4–6 subjects and 5 (16.7%) had clinical practice experience in 7 or more subjects.

### 3.2. Comparison of Handover Knowledge before and after the Program

The average level of handover knowledge was 4.63 ± 1.61 before the program and 5.83 ± 0.95 after the program, indicating a significant difference (t = −3.71, *p* = 0.001; Table 1).

### 3.3. Comparison of Self-Efficacy before and after the Program

The average self-efficacy rate was 3.35 ± 0.57 before the program and 3.90 ± 0.60 after the program, showing a significant difference (t = −5.65, *p* < 0.001; Table 2).

### 3.4. Comparison of Handover Performance Competency before and after the Program

The average handover performance competency was 1.75 ± 0.25 before the program and 2.37 ± 0.21 after the program, indicating a significant difference (t = −12.08, *p* < 0.001; Table 3).

## 4. Discussion

Despite the high necessity and effectiveness of handover education for nursing students, research on improving handover performance competency has thus far not been conducted. Therefore, this study developed and verified its effectiveness using a simulation-based handover education program. It was based on a scenario similar to an actual clinical environment, to enhance their handover capability before graduation and help them more easily adapt to clinical practice.

We evaluated nurses’ handover knowledge before and after a simulation-based handover education program and found a significant increase in knowledge level. This was similar to findings from Seada and Bayoumy [31], who surveyed nurses’ interns working in intensive care units to confirm the effectiveness of the handover education program. Thus, it verified that handover education is effective in improving knowledge that can be applied to clinical practice [15]. Nurses must be aware of handover’s effects on patient safety and develop a sense of responsibility and duty [18]. However, as the responsibility and duty of handover cannot be reflected in nursing scenarios, it is recommended that alternate education methods, such as lectures, be provided in addition to simulation-based education [32]. Thus, the handover education program in this study was designed to use both theoretical lectures and simulation-based education. Providing nursing students the opportunity to summarize and organize the contents of education through introspection and debriefing may have helped improve handover knowledge.

After comparing self-efficacy among nursing students before and after participating in the program, it was found to have significantly increased after the program. This is similar to the results of previous studies conducted with nurses that showed a significant increase in self-efficacy after implementing a communication analysis handover education program [33] and a communication education program based on scenarios [34]. Moreover, the results of the present study were in accordance with another study on senior-year medical students, in which confidence increased after attending a program that used handover-related videos and structured handover tools [35]. Another study reported that simulation-based education provided to students in a group might not increase self-efficacy, due to the importance of each member’s role [36]. In this study, education was provided to each student individually using a high-fidelity patient simulation. Thus, self-efficacy may have improved, as students completed the simulation process by themselves, from patient evaluation to handover, in a clinical-like environment. Thus, it is necessary to consider providing individual education when designing a simulation-based handover education program for nursing students.

Participants’ handover performance competency increased significantly after the program. This finding is in line with a previous study finding that clinical performance competency level increased more after simulation-based handover education than after small-group handover education for new nurses [21]. Moreover, this finding is also consistent with the study by Berkenstadt and colleagues [18] that found that nurses’ handover performance competency increased significantly through an education program emphasizing communication skills and simulation-based education. In other words, simulation-based handover education was effective in improving handover performance competency. In the present study, the handover education program implemented a realistic situation based on scenarios about caring for patients with respiratory problems. Students experienced performing an assessment, taking action, determining the content to hand over, and performing handover. This is significant, as the simulation reflected the workflow of patient handover in clinical settings. To establish a systematic education program to improve handover performance competency, it is necessary to conduct research that comprises a pool of different scenarios, develops ways for nursing students to experience not only assessment and action but also communication skills, and verifies the effects.

Although the type and time of handover varies according to the clinical setting and medical environment, most patient handovers are performed orally [7]. In terms of quality management, it is important for clinical nurses to perceive changes in the patient’s condition, conduct accurate assessments, handle the situation appropriately, determine significant results, and clearly communicate with other nurses. Thus, by expanding simulation training based on scenarios, which is currently implemented in many colleges, and linking it to handover, it will be possible to improve nursing knowledge and competencies and apply them to clinical practice. To maximize the effects of simulation-based handover education in this context, it is necessary to introduce and apply these strategies to regular training courses for senior-year nursing students who will soon be joining clinical practice.

### Limitations

There are two limitations to this study: First, because it was conducted with students belonging to only one women’s nursing college, the findings cannot be generalized to all nursing students or student populations. Second, as this study was conducted with a single-group pre- and post-design, the effect of the interventional design was not assessed by comparing it with a control group.

## 5. Conclusions

Patient handover is a type of indirect nursing care that requires high-level nursing competencies to make comprehensive clinical decisions. It was found that the simulation-based handover education program was effective in improving handover knowledge, self-efficacy, and handover performance competency in nursing students. Thus, this program can be used as a basis for establishing an education system to improve handover performance competency. Furthermore, by expanding the scope of application to new nurses, nurses who have not worked in clinical practice for long-term, and nursing students, handover quality and patient safety can be improved.

In this study, we developed a simulation-based handover educational program for nursing students and confirmed its effectiveness. In terms of nursing practice, this program was designed to allow nurses to experience handover after patient nursing, based on clinical cases. This program is expected to be a basis for establishing a future handover-education system for improving nursing education. In terms of nursing administration, if a fourth-grade nursing student uses a simulation-based handover educational program, they can more easily adapt to the patient handover task in a clinical setting in the future. Furthermore, it is expected that nurses’ performance and job satisfaction will be improved. Finally, this program can help improve the quality of nursing handover, contributing to patient safety.

## Figures and Tables

**Table 1:** Comparison of handover knowledge before and after the education program (*N* = 30).

Variables	Pre-Test M ± SD	Post-Test M ± SD	t	*p*
Handover knowledge	4.63 ± 1.61	5.83 ± 0.95	−3.71	0.001

**Table 2:** Comparison of self-efficacy before and after the education program (*N* = 30).

Variables	Pre-Test M ± SD	Post-Test M ± SD	t	*p*
Self-efficacy	3.35 ± 0.57	3.90 ± 0.60	−5.65	<0.001
I believe I can perform a handover whenever I plan to.	3.07 ± 0.91	3.77 ± 0.94	−3.25	0.003
I can perform a handover if I am required to.	3.27 ± 0.79	3.90 ± 0.76	−3.90	0.001
I can work hard even when it is difficult to start a handover.	3.37 ± 0.68	4.23 ± 0.73	−4.07	<0.001
I can accomplish the goal of a handover.	3.37 ± 0.72	3.83 ± 0.83	−2.84	0.008
I do not give up on any task and ensure I complete it.	3.20 ± 0.81	3.97 ± 0.67	−5.14	<0.001
I can attempt a difficult handover.	3.83 ± 0.75	4.20 ± 0.66	−3.27	0.003
I can attempt a complicated handover.	3.80 ± 0.71	4.27 ± 0.69	−3.75	0.001
I can perform an unpleasant handover if I am required to.	3.83 ± 0.79	4.20 ± 0.76	−2.63	0.014
I can perform a sudden handover if a patient has to be taken to the ward.	1.17 ± 0.59	3.30 ± 0.95	−11.22	<0.001
Even if I fail at first, I do not give up when learning something new.	1.00 ± 0.74	3.93 ± 0.74	−16.39	<0.001
I can handle unexpected events during a handover.	2.80 ± 0.81	3.60 ± 0.93	−4.25	<0.001
I try to learn even if the handover seems difficult.	4.13 ± 0.63	4.23 ± 0.63	−0.83	0.415
I am not frustrated when I fail to perform a handover and try to work even harder.	3.70 ± 0.88	4.20 ± 0.66	−2.92	0.007
I am confident about my ability to perform a handover.	2.70 ± 0.99	3.43 ± 1.01	−3.19	0.003
I trust myself to perform a handover.	2.93 ± 0.98	3.63 ± 0.96	−5.11	<0.001
I do not give up while performing a handover.	3.63 ± 0.72	4.03 ± 0.67	−3.25	0.003
I can handle any problems that may arise during a handover.	2.87 ± 0.78	3.57 ± 0.82	−3.53	0.001

**Table 3:** Comparison of handover performance competency before and after the education program (*N* = 30).

Variables	Pre-Test M ± SD	Post-Test M ± SD	t	*p*
Handover competency	1.75 ± 0.25	2.37 ± 0.21	−12.08	<0.001
Handover of the patient, room, and diagnosis	1.90 ± 0.31	2.53 ± 0.51	−5.64	<0.001
Perform a handover of a patient, room number, and diagnosis	2.23 ± 0.43	2.60 ± 0.56	−3.61	<0.001
Perform a handover after recognizing the patient’s primary complaint and objective outcome	2.20 ± 0.41	2.57 ± 0.57	−3.27	0.003
Perform a handover after recognizing abnormal changes in vital signs	1.77 ± 0.77	2.43 ± 0.63	−4.33	<0.001
Perform a handover of a meaningful situation such as a procedure, date, etc.	1.60 ± 0.56	2.37 ± 0.72	−4.89	<0.001
Perform a handover of meaningful drugs, treatment, and diet	1.07 ± 0.25	2.73 ± 0.58	−15.05	<0.001
Perform a handover of meaningful test results	1.00 ± 0.00	1.30 ± 0.47	−3.53	0.001
Explain about falls and their risks	2.30 ± 0.60	2.90 ± 0.40	−4.27	<0.001
Perform a handover of the reason for hospitalization	2.17 ± 0.59	2.57 ± 0.68	−2.56	0.016
Perform a handover of meaningful history	2.00 ± 0.74	2.40 ± 0.62	−2.18	0.037
Perform a handover of medicines taken, allergies, and other diseases specific to medication	1.70 ± 0.54	2.40 ± 0.56	−3.06	0.005
Perform a handover of the preparations and proceedings of tests to be conducted	1.63 ± 0.56	2.17 ± 0.70	−3.57	0.001
Perform a handover of an action to be taken	1.83 ± 0.70	2.37 ± 0.67	−3.25	0.003
Perform a handover of the situation of other parties	1.77 ± 0.82	2.50 ± 0.68	−4.10	<0.001
Perform a handover of a patient’s and caregiver’s needs	1.07 ± 0.25	1.77 ± 0.68	−5.11	<0.001

## Data Availability

The data presented in this study are available on request from the corresponding author. The data are not publicly available due to privacy.
